# Responsiveness, Reliability, and Minimally Important and Minimal Detectable Changes of 3 Electronic Patient-Reported Outcome Measures for Low Back Pain: Validation Study

**DOI:** 10.2196/jmir.9828

**Published:** 2018-10-24

**Authors:** Robert Froud, Carol Fawkes, Jonathan Foss, Martin Underwood, Dawn Carnes

**Affiliations:** 1 Clinical Trials Unit Warwick Medical School University of Warwick Coventry United Kingdom; 2 Institute of Health Sciences Kristiania University College Oslo Norway; 3 Barts and the London School of Medicine and Dentistry Queen Mary University of London London United Kingdom; 4 Department of Computer Science University of Warwick Coventry United Kingdom; 5 Faculty of Health University of Applied Sciences and the Arts Western Switzerland Switzerland

**Keywords:** electronic patient-reported outcome measures, validation, responsiveness, reliability, minimally important change, minimal detectable change, Roland Morris Disability Questionnaire, visual analog scale, numerical rating scale

## Abstract

**Background:**

The Roland Morris Disability Questionnaire (RMDQ), visual analog scale (VAS) of pain intensity, and numerical rating scale (NRS) are among the most commonly used outcome measures in trials of interventions for low back pain. Their use in paper form is well established. Few data are available on the metric properties of electronic counterparts.

**Objective:**

The goal of our research was to establish responsiveness, minimally important change (MIC) thresholds, reliability, and minimal detectable change at a 95% level (MDC_95_) for electronic versions of the RMDQ, VAS, and NRS as delivered via iOS and Android apps and Web browser.

**Methods:**

We recruited adults with low back pain who visited osteopaths. We invited participants to complete the eRMDQ, eVAS, and eNRS at baseline, 1 week, and 6 weeks along with a health transition question at 1 and 6 weeks. Data from participants reporting recovery were used in MIC and responsiveness analyses using receiver operator characteristic (ROC) curves and areas under the ROC curves (AUCs). Data from participants reporting stability were used for analyses of reliability (intraclass correlation coefficient [ICC] agreement) and MDC_95_.

**Results:**

We included 442 participants. At 1 and 6 weeks, ROC AUCs were 0.69 (95% CI 0.59 to 0.80) and 0.67 (95% CI 0.46 to 0.87) for the eRMDQ, 0.69 (95% CI 0.58 to 0.80) and 0.74 (95% CI 0.53 to 0.95) for the eVAS, and 0.73 (95% CI 0.66 to 0.80) and 0.81 (95% CI 0.69 to 0.92) for the eNRS, respectively. Associated MIC thresholds were estimated as 1 (0 to 2) and 2 (–1 to 5), 13 (9 to 17) and 7 (–12 to 26), and 2 (1 to 3) and 1 (0 to 2) points, respectively. Over a 1-week period in participants categorized as “stable” and “about the same” using the transition question, ICCs were 0.87 (95% CI 0.66 to 0.95) and 0.84 (95% CI 0.73 to 0.91) for the eRMDQ with MDC_95_ of 4 and 5, 0.31 (95% CI –0.25 to 0.71) and 0.61 (95% CI 0.36 to 0.77) for the eVAS with MDC_95_ of 39 and 34, and 0.52 (95% CI 0.14 to 0.77) to 0.67 (95% CI 0.51 to 0.78) with MDC_95_ of 4 and 3 for the eNRS.

**Conclusions:**

The eRMDQ was reliable with borderline adequate responsiveness. The eNRS was responsive with borderline reliability. While the eVAS had adequate responsiveness, it did not have an attractive reliability profile. Thus, the eNRS might be preferred over the eVAS for measuring pain intensity. The observed electronic outcome measures’ metric properties are within the ranges of values reported in the literature for their paper counterparts and are adequate for measuring changes in a low back pain population.

## Introduction

Low back pain is a common and costly problem resulting in substantial personal, social, and economic burdens and is the number one cause of disability globally [1,2]. Low back pain is a symptom rather than a disease and most low back pain is nonspecific (ie, where no specific underlying cause has been identified, but where the term lacks formal definition and where definitions in trials have been diverse) [1,3]. The lifetime prevalence of low back pain is between 60% and 84% [4,5]. The global problem of low back pain is getting worse due to aging and increasing population size [6,7]. The number of clinical trials of interventions for low back pain has been increasing, with over 30 trials of interventions for low back pain now being published annually [8]. Patient-reported outcome measures (PROMs) in the form of paper questionnaires are typically used in these trials to judge the effectiveness of the health technology under investigation [8].

Disability and pain are by far the most commonly measured domains in trials of interventions for low back pain; each is measured at least twice as often as any other domain [8]. The visual analog scale (VAS) and numerical rating scale (NRS) are most commonly used for measuring pain intensity and the Roland Morris Disability Questionnaire (RMDQ) is most commonly used for measuring functional disability [8]. These are quasi-continuous measures where the relationship between the observed item responses and the unobserved latent variable is assumed to be consistent with a reflective conceptual framework [9]. There is evidence that paper forms of VAS and NRS have been in use since at least the early to mid-20th century, and the RMDQ has been used since 1983 [10-12].

The validity of a PROM is defined as “the degree to which an instrument truly measures the construct(s) it purports to measure” [13]. Several aspects that compose what we consider to constitute good development and validation of PROMs postdate the introduction of these particular instruments. Validation exercises have been performed retrospectively, results have accrued over time, and endorsement and use of the measures have survived the process [14-16]. Notwithstanding healthy academic debate, it is generally accepted that these outcome measures have reasonable face validity and content validity, and they have at times been considered the legacy gold standard for comparison for assessing the criterion/convergent validity of other instruments [17-19].

Measuring patient/participant change in health status using browser-based technology and mobile device technologies is a natural progression. Digital PROMs and ports of existing paper PROMs to digital media have become known as electronic patient-reported outcomes measures [20]. When migrating existing paper PROMs to electronic patient-reported outcome measures (ePROs), there are aspects relating to the metric validity of the instrument that may need to be reassessed. Some aspects of validity are clearly independent of whether the instrument is completed on paper or digitally—for example, the content wording (unless it is culturally or clinically out of date) and the extent to which this content is judged to appropriately span the domains of the health construct being measured (ie, content and face validity). However, other aspects of validity that relate directly to measurement performance should not be assumed to be unchanged.

For any instrument designed to measure change in a health construct, 2 properties are particularly relevant: reproducibility (ie, reliability) and responsiveness. Reliability is the extent to which the same results are obtained on repeated measures when no real change in health status has occurred [21,22]. An analogy using a bathroom scale is that it is desirable that the scale shows the same weight upon time-standardized daily measurement when there truly is no true change in a person’s weight; if this is the case, the scale may be said to be reliable. Conversely, responsiveness is analogous to the scale detecting an important change when one truly exists. As users’ physical interactions with ePRO versions of PROMs differs in fundamental respects from paper versions, we suggest that reassessing these 2 key change measurement properties is necessary before advocating their widespread use in health research.

In analyses of trials or evaluations of health interventions, using PROMs to decide when an individual participant has responded facilitates interpretation of intervention effect [23]. Responder analysis permits the number of improvements to simply be counted and compared by arm using several clear statistics. These are intuitive reporting methods, and there is consensus that back pain trials should incorporate these [23-25]. However, to be able to do this, it is necessary to know (1) the minimum thresholds considered important to an individual participant—the minimally important change (MIC)—and (2) what magnitudes of change can be detected beyond the inherent measurement error of the instrument—the minimal detectable change (MDC) [26,27]. These thresholds may be altered by the change in media from paper to digital and may also be population specific [28,29].

We aimed to determine reliability and responsiveness, MIC and MDC, for electronic versions of the VAS, RMDQ, and NRS as administered via Web browser and Android or iOS app to adults with low back pain who visit osteopaths.

## Methods

### Recruitment

We recruited adults with low back pain from osteopathic clinics in England and Wales. Participants were recruited by osteopaths on our behalf and provided with an enrollment code and instructions for installing the iOS or Android app (from the App Store or Google Play) or completing the outcome measures using a Web browser.

We assumed an attrition rate of up to 70% and a recovery rate (ie, participants who indicate that they are much better or completely recovered using a health transition question) of over 90% in those with acute and subacute low back pain (ie, low back pain present for less than 3 months) [30]. Thus, for our responsiveness study, for which we required improved participants, we sought to recruit a minimum of 200 people with acute and subacute low back pain to ensure at least 50 eligible 6-week measurements. For people with chronic low back pain receiving manual therapy, we assumed up to the same rate of attrition but a lower rate of recovery (45%) [24]. For our test-retest study, we required stable participants who identified as remaining stable over a period of 1 week; thus, we sought to recruit 400 chronic patients to find 50 participants self-identifying as stable (ie, reporting no change on a health transition question). Participants were invited to complete the electronic versions of outcome measures at baseline, 1-week, and 6-week follow-up time points. Participants were offered a £5 (US $7) retail gift voucher for completing the outcome measures.

### Software

We used Android and iOS apps and a Web app with an associated form builder that was developed by Clinvivo Ltd, a University of Warwick spin-out company [31]. The apps, which function identically across platforms, permitted PROMs to be typeset and then administered to patients securely on their own devices. Data in transit are encrypted using a Secure Sockets Layer, and data at rest are encrypted using a Rivest-Shamir-Aldeman and Advanced Encryption Standard encryption hybrid. At the end of the study period, data were encrypted using the open Pretty Good Privacy standard and transferred from Clinvivo to researchers. The iOS, Android, and Web apps sent data one way and did not receive or redisplay personal data. The platform presented an electronic version of the instrument and reminded participants to complete outstanding follow-up measurements, as appropriate. Off-line completion in apps was permitted in cases of interrupted connectivity, with submissions occurring upon restoration of connectivity. Reminders, which were received up to twice per follow-up measurement due, were sent directly to devices for app-enrolled participants and by email to Web-enrolled participants (up to 2 reminders).

### Electronic Versions of Patient-Reported Outcome Measures

**Figure 1:**
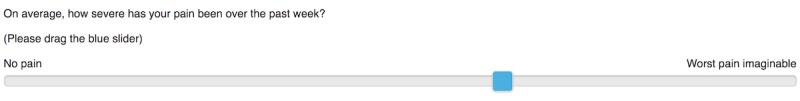
Electronic visual analog scale for pain intensity showing 63 units of pain intensity.

The VAS is a continuous scale running from 0 to 100 mm measuring current pain intensity [32]. It is the most commonly used outcome measure in trials of interventions for nonspecific low back pain overall [8]. Huskisson is commonly credited with its development in 1974; however, there is evidence that it was being used at least as far back as 1921 [11]. Intellectual property rights are in the public domain, and no permissions are required for use, reproductions, or modifications. Completion of the paper scale involves a person marking a line on the scale indicating their level of pain between 2 anchored scales that typically have wordings of “no pain” on the left (ie, 0 mm) and “worst possible pain” or “worst imaginable pain” on the right (ie, 100 mm) [33,34]. On paper, the distance of the marked line is then measured from the point of 0 pain and reported in mm. In migrating this to an electronic version (eVAS), we implemented a slider that could be dragged into position. We did not force the scale to render at 10 cm to allow for resizing to screens of different devices. Thus, we report scores in units rather than mm, where 1 unit is 1/100th of the scale (ie, where the pointer can be set at any one of 101 different positions) as rendered (Figure 1).

**Figure 2:**
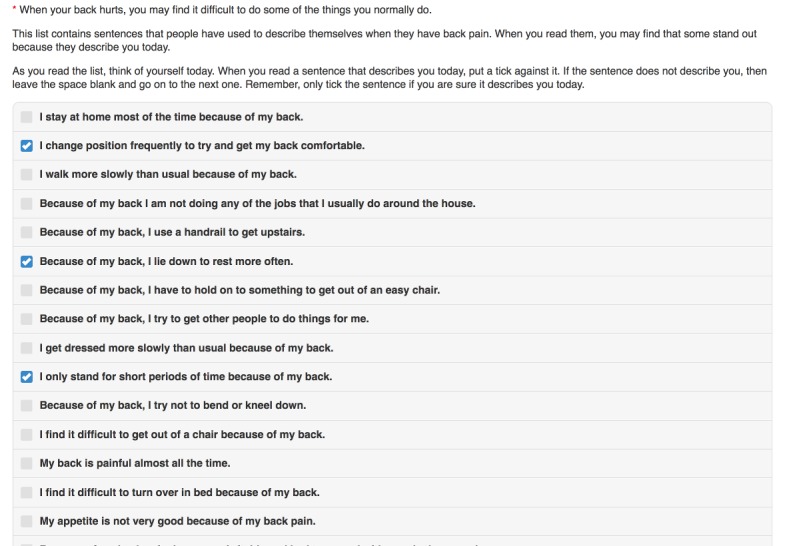
Electronic Roland Morris Disability Scale showing a part score of 3 units.

The RMDQ is a 24-item questionnaire measuring functional disability due to back pain that was developed in the early 1980s [10]. It is the most commonly used outcome measure in trials of interventions for low back pain overall [8]. The original paper version of the instrument is well established [35-38]. No permissions are required for its use, reproductions, or modifications [39]. Scores on the RMDQ range from 0 to 24, where higher scores indicate greater disability. Participants are given a statement with which they may indicate agreement by ticking a box. Participants are asked to tick statements that they feel describe them on that day and to leave blank boxes next to statements that they feel do not. The score is then the sum total of checked items. Our electronic (eRMDQ) migration is an exact copy using multiselect check boxes (Figure 2). One year into the research, we added a box stating “none of the above symptoms” for participants to confirm that none of the statements applied to them and to confirm 0 scores were genuine and not reflective of a skipped question.

**Figure 3:**
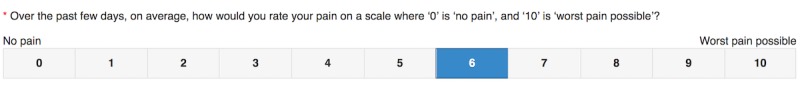
Electronic numerical rating scale for pain intensity showing a part score of 6 units.

The NRS is an 11-point ordinal scale measuring current pain intensity [40,41]. Validation of the paper version is well established [41-43]. It is the fourth most commonly used outcome in trials of interventions for low back pain overall [8]. It is well established, with intellectual property rights in the public domain. Scores on the NRS range from 0, which typically is anchored “no pain” and 10, which typically is anchored “worst pain possible.” Our electronic (eNRS) migration is an exact copy with these anchor wordings (Figure 3). As the range of responses is exhaustive, completion of the scale was required for submission.

Participants were also asked to electronically complete a health transition question at 1- and 6-week follow-up time points. The transition question was a single question with the wording “Overall, how would you rate the change in your symptoms since beginning this study?” where the participant could respond on a 7-point scale [44]: 1–completely recovered, 2–much improved, 3–slightly improved, 4–no change, 5–slightly worsened, 6–much worsened, and 7–vastly worsened.

### Assessment

We aimed to have 50 completed paired measurements in improving participants for responsiveness assessments and 50 completed test-retest measurements in stable participants. We defined improving participants a priori as participants who selected much improved or completely recovered using the transition question. Improving participant scores were used to assess responsiveness at 1 and 6 weeks. For our test-retest study, we defined stable participants a priori as those who select no change at 1 week, and in the case of having too few observations, a post hoc sensitivity analysis including those who selected either slightly worsened, no change, or slightly improved. This alternative “about the same” approach to marking stability has been used elsewhere [45]. Allowing 1 week is typical in low back pain test-retest studies; clinically, this is close enough for the people with chronic pain to remain stable but far enough apart that participants cannot easily recall their initial responses. It was anticipated that the chronic population would predominantly contribute participants to the test-retest study and improving participants would come from across all chronicity subpopulations.

### Statistical Analyses

To measure responsiveness in a way that is consistent with the Consensus-Based Standards for the Selection of Health Measurement Instruments (COSMIN) definition, we constructed receiver operator characteristic (ROC) curves for 1- and 6-week data using a dichotomized transition question as the external criterion [22]. The area under the ROC curve (AUC) is then a metric of responsiveness, accepting that the external criterion reasonably includes the construct of interest [46]. The approach has previously been used to quantify responsiveness across all 3 paper versions of instruments [47]. ROC AUCs of over 0.70 were considered to be adequate [9,48]. We dichotomized the transition question such that participants responding completely recovered and much improved were considered improved and all other responses were considered not improved.

We also used ROC curves and the transition question external criterion for 1- and 6-week data to quantify the MIC, which is defined as “the smallest [change] in score in the domain of interest which patients perceive as beneficial and which would mandate, in the absence of troublesome side effects and excessive cost, a change in the patient’s management” (see note 1 in Multimedia Appendix 1) [43,49]. We used a MIC estimator based on the minimum sums of squares method, which consistently selects the cut-point closest to the top left corner of ROC space, as required when sensitivity and specificity are valued equally [50]. We calculated confidence intervals for MIC point estimates using bootstrapping [51].

To estimate reliability, we calculated intraclass correlation coefficients (ICCs) [52,53]. ICC values usually range from 0 to 1 [54]. ICC values above 0.75 may be interpreted as excellent agreement, values of 0.40 to 0.75 indicate poor to fair agreement, and values of below 0.40 indicate poor agreement [55]. We calculated the standard error of measurement [53]. We used this to estimate the minimal detectable change at the 95% level (MDC_95_) (see notes 2 to 4 in Multimedia Appendix 1) [53,56,57].

Transition questions can be highly correlated with follow-up score rather than change [24,43,58]. Guyatt et al [58] assert that if a transition question is truly measuring change then a correlation between the baseline score and transition question and the follow-up score and transition question should ideally be present, equal, and opposite. In addition, they suggest that in a linear regression model with follow-up score entered as the initial explanatory variable, the baseline score should explain a significant proportion of the residual variance in the transition rating [58]. We performed Pearson correlations and fitted regression models to explore the degree to which the transition question measured change or simply reflected follow-up status. Log rank tests were used to assess significance of the addition of baseline score.

All analyses were performed using Stata version 14.2 (StataCorp LLC). The program rocmic was used to estimate MIC and the ROC AUC, which for ROC AUC uses the lroc program [51,59].

### Power and Sample Size

With the notable exception of construct validity, sample sizes in validation studies generally are not calculated based on power to test hypotheses: the estimation of reliability and responsiveness parameters is focused on the extent to which the coefficients describing these parameters approach 1 (which would represent perfect reliability/responsiveness) rather than their difference from 0 or some other null value. Generally, a sample size of at least 50 participants is considered adequate for this purpose [9,60]. Assuming an ICC of 0.7, with 50 participants we would be able to estimate the ICC to within a 95% CI of +/–0.14. Alternatively, for an ICC of 0.8, we would be able to estimate to within a 95% CI of +/–0.10 [9]. For responsiveness, with 50 participants and assuming an AUC of 0.8 and equal numbers of cases and noncases, we would be able to estimate AUC to within a 95% CI of +/–0.12 [61].

As standard errors (SEs) for MIC estimates are not readily calculable, we used bootstrapping to generate SEs and 95% CIs [51,62]. Previous simulation work on the paper-based RMDQ in a similar population suggested that 2500 bootstrap samples was sufficient to ensure SE convergence [63]. To explore whether this is the case for the eRMDQ (and also whether it is an appropriate number of replications for the eNRS and eVAS), we simulated SEs by randomly sampling n observations (with replacement) from our dataset for an increasing number of n, where n is an integer, beginning at 20 and increasing by increments of 20, up to 6000 [62,64]. We then graphically assessed SE convergence and used the point of convergence to inform the number of bootstrap replications.

### Data Exclusions, Assumptions, and Variations

Prior to the addition of the “none” box, we imputed 0 scores for all baseline submissions with no eRMDQ boxes ticked and assumed and imputed a 0 score for eRMDQ follow-up scores in the case that the baseline eRMDQ score was greater than 0 and a submission had been made for the follow-up period in question. When the eVAS rendered, it did so with the slider in the 0 position. In the case of a submission for an untouched eVAS, a score of 0 was assumed valid. The eNRS was a required response and necessitated a selection for submission.

As part of the basic demographic details collected, we included a list of presenting complaints, featuring low back pain among 15 other common musculoskeletal presentations and the opportunity to report a complaint not listed in a free-text box. The list of complaints was derived from earlier survey work developed as part of a national data collection initiative [65,66]. We excluded all cases where a participant had not checked the low back pain box (data from non-low back pain cases were used in unrelated research).

### Ethics Approval

Ethics approval was obtained from the research ethics committee at Queen Mary University of London (QMERC2014/18).

## Results

### User Statistics and Demographics

**Figure 4:**
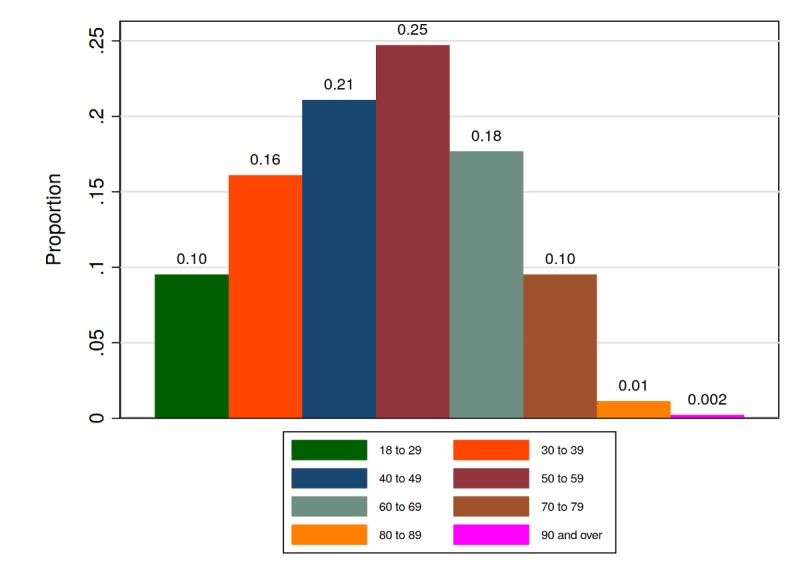
Histogram of patient age at baseline.

We collected data from 575 people from 30 osteopathic clinics between July 15, 2014, and May 3, 2017. Of these, 442 (76.9%) reported low back pain as their main complaint. The average submission time for 1-week scores was 7.4 (SD 0.79) days after baseline. The average submission time for 6-week scores was 42.5 (SD 0.9) days after baseline. Of the participants, 60.4% (267/442) were female, 69.2% (306/442) identified as being in full or part-time employment, 1.1% (5/442) were long-term sick, 3.6% (16/442) identified as looking after home/family, 19.7% (87/442) were retired, 1.4% (6/442) were in full-time education, 2.9% (13/442) were unemployed, and 2.0% (9/442) selected other or preferred not to disclose. Figure 4 shows a histogram of patient-reported age at baseline.

**Figure 5:**
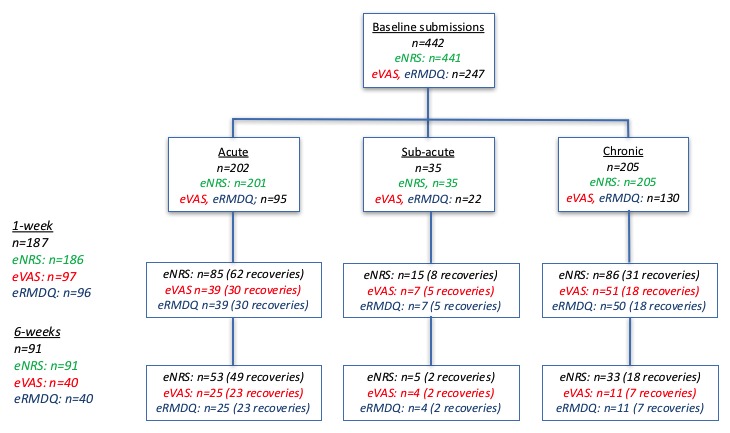
Flowchart showing completion rates at 1 and 6 weeks, chronicity status, and the incidence of self-reported recovery using the health transition question for participants who also completed the electronic numerical rating scale, and electronic Roland Morris Disability Questionnaire, and electronic visual analog scale measurement.

We collected baseline eNRS data from 442 participants, and we collected baseline eVAS and eRMDQ data from 247 participants. One-week data were collected from 187 and 97 participants, respectively, and 6-week data were collected from 91 and 40 participants, respectively. Figure 5 shows the incidence of recovery in these groups. There was 1 missing data point for eNRS at baseline (0.2%) and 1 week (0.5%) for which we were unable to confirm cause. Table 1 summarizes ePRO submission scores using median and interquartile range and Table 2 summarizes recoveries and cumulative recoveries recorded using the transition question. Change scores (not shown) more closely followed normal distributions.

The addition of baseline score generally explained a significant proportion of the variance in the transition question over and above follow-up score. The transition question correlated with follow-up score but not with baseline score. Comprehensive results for the Guyatt analyses on the transition question’s performance in measuring change are listed in note 2 in Multimedia Appendix 1.

### Evaluation Outcomes

**Figure 6:**
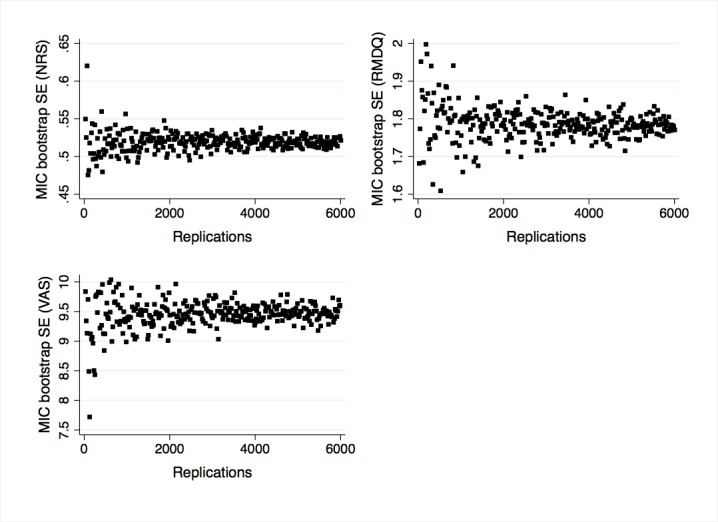
Graphs showing minimally important change bootstrap standard error convergence from simulations with increasing replication numbers. MIC: minimally important change, NRS: numerical rating scale, RMDQ: Roland Morris Disability Questionnaire, VAS: visual analog scale.

Graphically, SE convergence appeared to be asymptotically complete at around 5000 bootstrap replications (Figure 6); thus 5000 replications were used to generate confidence intervals for the MIC estimates in Table 3. Responsiveness point estimates (Table 3) were borderline adequate (AUC≈0.7) or above adequate for all instruments and time points. The AUC confidence interval for the RMDQ at 6 weeks spanned the null value (Table 3).

Using no change as a criterion for judging stability, we did not achieve our a priori threshold of 50 test-retest data points for comparison across any of the instruments. Of the people who said they had no change at 1 week, 65% (15/23) had chronic pain. Allowing slightly improved and slightly worsened to count as stable enabled us to achieve this threshold for the eNRS only. Of people who said they had no or slight change at 1 week, 63% (53/84) had chronic pain. Notwithstanding the lack of data, the eRMDQ reliability (agreement) was excellent using either analysis, with CIs spanning fair to excellent in both analyses (Table 4). For the eVAS per protocol analysis, the agreement was fair with CIs spanning poor to fair, and in the sensitivity analysis, the agreement was poor to fair with a CI range spanning poor to fair (Table 4). For the eNRS per protocol analysis, the agreement was poor to fair with a CI spanning poor to excellent, and for the sensitivity analysis, agreement was fair with a CI spanning poor to fair to excellent (Table 4).

**Table 1:** Baseline, 1-week, and 6-week scores across the whole sample.

Score	Baseline		1 week		6 week	
	Median (IQR)	n^a^	Median (IQR)	n	Median (IQR)	n
eRMDQ^b^	4 (6)	247	2 (4)	96	2 (3.5)	40
eVAS^c^	41 (32)	247	24 (19)	97	19 (19)	40
eNRS^d^	5 (4)	441	3 (3)	186	2 (2)	91

^a^The number of received measurements at 1 week and at 6 weeks, respectively.

^b^eRMDQ: electronic Roland Morris Disability Questionnaire.

^c^eVAS: electronic visual analog scale.

^d^eNRS: electronic numerical rating scale.

**Table 2:** Recoveries and cumulative recoveries recorded using the transition question

Transition question	n	Recoveries, n (%)	Cumulative recoveries, n^a^ (%)
1 week	187	101 (54)	101 (23)
6 weeks	91	69 (76)	170 (38)

^a^Where the frequency of cumulative recoveries are shown as a proportion of all 442 baseline participants.

**Table 3:** Responsiveness and minimally important change by instrument and 1-week and 6-week follow-up time periods.

Instrument and time period		Receiver operator characteristic AUC^a^	95% CI	n^b^	Minimally important change points/ eVAS^c^ units (% of baseline score)	95% CI
**eRMDQ^d^**							
	1 week	0.69	0.59 to 0.80	96	1 (19)	0 to 2
	6 weeks	0.67	0.46 to 0.87	40	2 (38)	–1 to 5
**eVAS**							
	1 week	0.69	0.58 to 0.80	93	13 (32)	9 to 17
	6 weeks	0.74	0.53 to 0.95	40	7 (17)	–12 to 26
**eNRS^e^**							
	1 week	0.73	0.66 to 0.80	185	2 (43)	1 to 3
	6 weeks	0.81	0.69 to 0.92	91	1 (21)	0 to 2

^a^AUC: area under the curve.

^b^The number of change scores available (ie, from available pairs of measurements at baseline and follow-up time point) at 1 week and 6 weeks, respectively.

^c^eVAS: electronic visual analog scale.

^d^eRMDQ: electronic Roland Morris Disability Questionnaire.

^e^eNRS: electronic numerical rating scale.

**Table 4:** Intraclass correlation coefficients from test-retest study in a per protocol stable sample and a pseudo-stable sample with associated minimal detectable change thresholds.

Instrument and condition		n^a^	Intraclass correlation coefficient_agreement_	95% CI	MDC_95_^b^ points/eVAS^c^ units
**eRMDQ^d^**						
	Per protocol	15	0.87	0.66 to 0.95	4
	Allowing slight change	43	0.84	0.73 to 0.91	5
**eVAS**						
	Per protocol	15	0.31	–0.25 to 0.71	39
	Allowing slight change	43	0.61	0.36 to 0.77	34
**eNRS^d^**						
	Per protocol	22	0.52	0.14 to 0.77	4
	Allowing slight change	83	0.67	0.51 to 0.78	3

^a^The number of cases satisfying the condition for analysis as a stable case.

^b^MDC_95_: minimal detectable change at the 95% level.

^c^eVAS: electronic visual analog scale.

^d^eRMDQ: electronic Roland Morris Disability Questionnaire.

^e^eNRS: electronic numerical rating scale.

## Discussion

### Principal Findings

The results suggest that the eRMDQ had borderline adequate responsiveness and excellent reliability. Conversely, the eNRS had relatively good responsiveness at 6 weeks but borderline adequate reliability. The eNRS outperformed the eVAS, which had adequate responsiveness but relatively poor reliability. As test-retest numbers were few, eVAS CIs spanned poor to excellent, and thus further investigation is warranted. While exploring use by age was not a specific study objective, we note the results indicate encouraging use by older people from this population.

### Comparison With Prior Work

Across acute and chronic back pain populations there has been like-for-like evaluation (ie, using similar and directly comparable methods) of the properties of paper versions of the outcome measures explored. ROC AUC for the RMDQ ranges from 0.64 to 0.93 [45,47,67-75]. ROC AUC for the NRS ranges from 0.67 to 0.93 [41,42,47,67,75,76]. ROC AUC for the VAS ranges from 0.71 to 0.93 [47,72,77-79]. Our results are within these ranges at 6 weeks for all instruments and for all but our eVAS instrument at 1 week, where our point estimate approaches the lower border of the range. Our eVAS data are nevertheless consistent with the range (ie, insofar as the upper CI overlaps). Estimates of ROC AUC for the VAS are fewer in the literature, which might explain why the range of reported results is narrower than it is for the RMDQ and NRS.

MIC thresholds for RMDQ ranged between 1.5 and 5.0 [21,24,35,67,68,72,75,80-83], for the NRS between 1.5 and 4.0 [41-43,67,75,81,84], and for the VAS between 15 and 28 mm [72]. Our absolute MIC thresholds are comparable but are toward the lower side of this range. MIC estimates are known to increase with baseline severity, and relatively low baseline scores likely explain our relatively low thresholds [68,75,81,84]. However, MIC thresholds in our results, expressed as percentage change from baseline, average 28% across all 3 instruments and all time points. This is consistent with the suggestion of Ostelo et al [29] (following their review of MIC and MDC literature) for using an improvement of between 20% and 30% of baseline score for the RMDQ, NRS, and VAS as a MIC threshold. We emphasize that the MIC thresholds relate to the degree of change that may be considered important for an individual and not what degree of difference may be considered important at a population level [27,85,86]. We note that the 2 negative CIs imply consistency of the data, with the true MIC thresholds being in the opposite direction of improvement (ie, a slight deterioration). This is likely an artifact of low power, and we suggest using inflated sample sizes for future studies based on the bootstrapped standard error observations.

Reported ICC estimates for the RMDQ have ranged from 0.42 to 0.95 [45,67,81,87] and for the NRS from 0.92 to 0.98 [67,81], and an estimate for the VAS of 0.71 has been reported [88]. Our results are within the ranges reported, but our ICC point estimate for the eVAS is lower than the reported paper VAS estimate. It is conceivable that rendering the eVAS slider in a 0 position might lead to additional variance in the case that the outcome is overlooked (ie, leading to a comparatively lower ICC), and future research might explore whether a touch to confirm 0 design is acceptable to users. We also note that some of the ICC values in the literature ranges may have been derived from ICCs for consistency rather than agreement; this is a practice known to exist (although it is not always clear which approach has been used) and known to overestimate reliability [53].

MDC_95_ estimates reported (or in the case of the NRS only, either reported or calculated from reported standard error of measurements) have ranged from 5.0 to 12.1 for the RMDQ [21,24,35,45,56,67,81,83], from 2.4 to 11 (ie, almost the full width of the scale) for the NRS [41,45,67,81,84], and from 21.0 to 33.5 for the VAS [79,88,89]. Our estimates are slightly better than average for the RMDQ, toward the lower end of the range for the NRS, and comparable to the available estimates for the VAS.

In terms of comparison to studies assessing these instruments as ePROs, Bird et al [90] conducted a test-retest study among 22 healthy adults of the VAS administered on a tablet and found ICCs of 0.90 (0.82 to 0.95) as compared to 0.96 (0.92 to 0.98) in a paper version that participants completed simultaneously. It is difficult to compare the results with this study, as the time between test and retest was less than 30 minutes. A much shorter period between test and retest might be appropriate in some populations (eg, where change in acute pain must be measured over short spaces of time). In these cases, participants may be more prone to panel conditioning, where the second response is affected by recall of the first response [91]. For back pain, most interventions focus on chronic pain and longer time periods. When exploring reliability of low back pain outcome measures, a 1-week gap between test and retest is typical. Bijur et al [92] and Gallagher et al [93] have used small time frames between tests on a paper-based VAS in acute pain populations and demonstrate similarly high ICCs of 0.97 (0.96 to 0.98) and 0.99 (0.989 to 0.992), respectively. Also of relevance but not directly comparable is work by Bishop et al [94], who administered the RMDQ on paper and online and constructed limits of agreement, demonstrating equivalence with a score difference of only 0.03 points and a Bland-Altman range of –2.77 to 2.83.

Finally, we note that the distribution of the user age of the health outcomes app in this population appears to be higher than the age of health app users [95].

### Implications

None of our results differs materially from ranges observed in population-similar and methodologically alike studies of paper counterparts. There is thus some suggestion that the ePROs under evaluation are suitable substitutes for PROMs for measuring change in low back pain. The eNRS outperformed the eVAS in terms of responsiveness and reliability. As such, we suggest the eNRS might be preferred over the eVAS for the measurement of low back pain intensity, but we caution that subsequent confirmatory research is warranted.

### Limitations

The principal limitation is that in several cases we had small sample sizes. We had intended to recruit sufficient numbers to have at least 50 people for each assessment, in line with recommendations, but we failed to meet these targets, mainly as we underestimated the incidence of stability, although we also underestimated attrition [9]. There were high rates of improvement in people receiving treatment, and this is a hazard of nesting a test-retest design within a protocol where participants are receiving routine clinical treatment. This was of consequence in the eRMDQ responsiveness analysis, where the data are consistent with a null population parameter and thus 6-week responsiveness of the eRMDQ requires confirmation in a larger sample. Having too few data has greater implications for the test-retest assessment of the VAS where the CIs span coefficient values that can be interpreted at their extremes as either poor or excellent. It is less of an issue for the eRMDQ because while the numbers are low and lower at 1 and 6 weeks, respectively, the stronger signal combined with boundary proximity leads to narrower and more useful CIs.

It is not ideal that we permitted slightly worse and slightly improved categories to indicate stability in our test-retest, although we note a similar approach has been observed previously [45]. Further, this was a post hoc decision taken in light of having too few observations to use our more stringent a priori criterion of including only those reporting no change. The results using our a priori approach but with few observations are offered as sensitivity analyses that may provide useful comparison.

Having relatively few observations also meant that we were unable to explore differences by platform (ie, iOS, Android, and Web browser) or explore MIC as a function of baseline score (eg, stratifying by number in category of severity) or separately by chronicity, which may have been useful and allowed us to explore any differences in these metrics by chronicity. Thus, our focus here is pragmatic and results are generalizable to the population of adults with low back pain who consult osteopaths, notwithstanding chronicity.

We recorded in our database only the summed eRMDQ score rather than individual responses. Had we retained detail of individual response profiles of the eRMDQ, we could have also calculated internal consistency (as well as aspects of modern test theory: Rasch analysis to examine item performance or factor analysis to explore data dimensionality). Whereas COSMIN conflate internal consistency with reliability in their taxonomy [22,96], we consider internal consistency to be an indication of the unidimensionality of a scale and of item redundancy rather than the degree to which a scale is free from measurement error. As such, and with respect to the reliability definition, we preferred to consider it separately. We had not immediately considered that the media used for completion might affect internal consistency or item functioning of a scale. On reflection, however, we think that it is conceivable that presenting the scale digitally may alter the way patients respond in such a way that these could be affected. Additionally, there may be self-selection effects of those more familiar with digital media joining the study, and this may be a factor that could be confounded with how a person responds.

It is not ideal that our transition question correlates with follow-up score but not with baseline score. This is emerging to be the case generally and is not something particular to evaluating electronic outcome measures [24,43,58]. This emergence in our view raises the more general question of whether it is appropriate to use transition questions at all to evaluate change in outcome measures. Apart from being overly driven by follow-up score, the assumption that the transition question is sufficiently driven by the same latent construct as the PROM, to the extent that it may be considered a gold standard, may be unrealistic. We have previously explored what people think about when they complete the transition question and what they think about when they complete the paper RMDQ version, and we found discordance [97]. Pain appears to be a greater driver of the transition question, and the wording of the transition question (ie, attempting to place focus specifically on function or an explicit domain) does not appear to matter. In our study, we used the term symptoms. However, in the case that the suggestion arising from our previous research is incorrect, using a generic wording in the transition question might have the advantage of not favoring any one ePRO over another but the disadvantage of disassociating the transition question from any specific latent health construct. Use of a generically worded transition question would then introduce some information bias—for example, if people systematically attend more to a particular domain upon reading the word symptoms. We caution that the logic of the typically taken approach of using one outcome measure as a proxy gold standard of recovery and then using this proxy to judge domain-specific responsiveness and MIC thresholds in another may be questionable where there is domain mismatch.

There was a small amount of missing data at baseline and 1 week (a person in each case), which should have been impossible because a selection on the eNRS was a required response. We are uncertain of the cause but we suspect this might have been due to use of an obscure and/or obsolete browser.

This research was conducted solely in private care and people who pay to see osteopaths may differ from those attending publicly funded health care, as is more routinely the case in health services research. We note a lower than typical baseline severity (as compared to clinical trials) and thus some caution is indicated before generalizing to typical trial populations. Finally, our focus here was on the most commonly used domains and outcome measures in trials. The VAS is most commonly used overall (pain), RMDQ second most common (disability), and the NRS fourth most commonly used (pain). We did not include the third most commonly used outcome, the Oswestry Disability Questionnaire, which also measures disability [8]. Unlike the VAS and NRS, which are both single-item instruments, including two full disability questionnaires risked being unduly burdensome for participants. Qualitative work suggests that participants would prefer to spend only 5 to 10 minutes completing ePROs [98,99]. Including a direct comparison with paper versions would have permitted direct exploration of criterion validity; however, this approach would likely have been affected by panel condition and further added to participant burden.

### Recommendations for Future Research

Sampling stable participants from people receiving routine clinical treatment allows the nesting of a test-retest design and makes for an efficient design. However, it produces some challenges for achieving sufficient recruitment over a realistic time period. It assumes that the transition question classification of unchanged is valid. As data suggest that transition question is driven more by follow-up state than change, the approach has some limitations. It would be scientifically preferable that test-retest studies are conducted within untreated populations. However, this has ethical and practical implications. When planning to nest a test-retest design within any treatment-containing protocol, based on rates observed in this study (using the lower eNRS no chance incidence), we recommend planning a study that is around 3 times larger (ie, seeking approximately 1200 people to obtain 50 stable participants). For study of responsiveness alone, about 250 participants should be sufficient to achieve 50 improvements at 6 weeks. The most extreme MIC threshold we estimated was 7 units (–12 to 26) for the eVAS at 6 weeks. This is lower than has been noted in studies of paper counterparts. Assuming the point estimate is representative of the population parameter, approximately 300 participants would be required to power a study to confirm the finding.

Retaining data at item level in future studies will permit more sophisticated analytics. There may need to be a cultural change as we transition from paper to digital measurement. The ability to more easily retain greater data resolution is a clear advantage of digital measurement and one that would be sensible to exploit. Further advantages in terms of cost, logistics, form validation, reminders, time logging, environmental factors, and reach are undeniable and, in our view, make electronic health measurement very attractive. More generally, routine outcome measurement in clinical practice may facilitate so-called learning health care systems and should be a shared goal of stakeholders across health care [100,101]. To achieve this, greater collaboration may be needed between clinicians, informatics specialists, and policy makers. We also encourage further metric testing of electronic versions of these and other legacy PROMs so that results may inform health services researchers and clinicians’ choices of measure.

### Conclusion

Each of the electronic outcome measures has metric properties that do not materially differ from values reported in the literature for their paper counterparts. A possible exception may be the reliability of the eVAS, for which there is insufficient existing research to make useful comparisons between paper and digital versions. The eRMDQ is adequate for measuring back-related disability, and the eNRS is adequate for measuring pain intensity. The eNRS should be preferred over the eVAS for the measurement of pain intensity.

## Appendix

Multimedia Appendix 1 Technical notes and extended technical results for Guyatt analyses.

## Abbreviations

AUC: area under the curve
COSMIN: Consensus-Based Standards for the Selection of Health Measurement Instruments
eNRS: electronic numerical rating scale
ePRO: electronic patient-reported outcome measure
eRMDQ: electronic Roland Morris Disability Questionnaire
eVAS: electronic visual analog scale
ICC: intraclass correlation coefficient
MIC: minimally important change
MDC: minimal detectable change
MDC _95_: minimal detectable change at the 95% level
NRS: numerical rating scale
PROM: patient-reported outcome measure
RMDQ: Roland Morris Disability Questionnaire
ROC: receiver operator characteristic
VAS: visual analog scale
