# Supplementary material for: Responsiveness, Reliability, and Minimally Important and Minimal Detectable Changes of 3 Electronic Patient-Reported Outcome Measures for Low Back Pain: Validation Study
Source: J Med Internet Res. 2018 Oct 24;20(10):e272. doi: 10.2196/jmir.9828 (PMC6231814; doi:10.2196/jmir.9828)
Supplement: Multimedia Appendix 1 [file jmir_v20i10e272_app1.pdf]

Technical appendix notes to accompany paper entitled ‘Responsiveness, Reliability, and Minimally Important and Minimal Detectable Changes of 3 Electronic Patient-Reported Outcome Measures for Low Back Pain: Validation Study’ by Froud *et al*

Note number	Note details
1	In the original definition, the Jaeschke *et al* use the word ‘difference’. However, we support the suggestion of de Vet *et al* of preserving the term ‘difference’ for discussing differences between groups or populations, and ‘change’ for discussing longitudinal within-group or within-person change. The Jaeschke definition relates to minimally important *individual change*.
2	ICC used accounting for systematic error (*i.e.* absolute agreement; Shrout and Fleiss ICC type 2.1),[52] which de Vet *et al* show is appropriate when measuring changes in patient health domains using quasi-continuous outcome measures.[53]
3	The square root of the sum of the ICC variance components for within-person and error variance. [53]
4	MDC_{95} was estimated as 1.96 x √2 x *SEM.* The MDC_{95} may be interpreted as the threshold equal to and below which the scores of participants drawn from a stable population may be considered to have a 95% probability of no real change having occurred. [53, 56, 57]
5	Baseline eVAS scores did not correlate with transition question scores at one-week (-0.04, P=.66) or at six-weeks (-0.12, *P*=.48), whereas follow-up scores correlated positively (0.32, *P*=.001) at one week (0.49, *P*=.001) at six-weeks. The addition of baseline score to follow-up score regressed on the transition question increased the variance explained from 11% to 16% (*P*=.01) at one-week and 24% to 30% at six weeks (*P*=.07). Baseline eNRS scores did not correlate with transition question scores at one-week (0.05, *P*=.49) or at six-weeks (-0.07, *P*=.52), whereas follow-up scores correlated positively (0.45, *P*<.001) at one-week (0.55, P<.001) at six-weeks. The addition of baseline score to follow-up score regressed on the transition question increased the variance explained from 20% to 25% (*P*<.001) at one-week and 30% to 31% at six weeks (*P*=.04). Baseline eRMDQ scores did not correlate with transition question scores at one week (0.04, *P*=.70) or at six weeks (-0.15, *P*=.35), whereas follow-up scores correlated positively (0.38, *P*<.001) at one week (0.34, *P*=.03) at six-weeks. The addition of baseline score to follow-up score regressed on the transition question increased the variance explained from 13% to 21% (*P*=.003) at one-week but did not significantly increase the variance at six-weeks (*P*=.16).
